# Exogenous Therapeutics of Microrna-29a Attenuates Development of Hepatic Fibrosis in Cholestatic Animal Model through Regulation of Phosphoinositide 3-Kinase p85 Alpha

**DOI:** 10.3390/ijms21103636

**Published:** 2020-05-21

**Authors:** Ya-Ling Yang, Feng-Sheng Wang, Hung-Yu Lin, Ying-Hsien Huang

**Affiliations:** 1 Department of Anesthesiology, Kaohsiung Chang Gung Memorial Hospital and Chang Gung University College of Medicine, Kaohsiung 833, Taiwan; inr453@cgmh.org.tw; 2 Core Laboratory for Phenomics & Diagnostics, Department of Medical Research, Kaohsiung Chang Gung Memorial Hospital and Chang Gung University College of Medicine, Kaohsiung 833, Taiwan; wangfs@ms33.hinet.net; 3 Department of Internal Medicine, Kaohsiung Chang Gung Memorial Hospital and Chang Gung University College of Medicine, Kaohsiung 833, Taiwan; 4 Center for Mitochondrial Research and Medicine, Kaohsiung Chang Gung Memorial Hospital and Chang Gung University College of Medicine, Kaohsiung 833, Taiwan; 5 Department of Pediatrics, Kaohsiung Chang Gung Memorial Hospital and Chang Gung University College of Medicine, Kaohsiung 833, Taiwan

**Keywords:** microRNA-29a, hepatitis, fibrosis, therapeutics, PI3KP85α, mitochondrial unfolded protein response, proteostasis

## Abstract

Recent studies have found that microRNA-29a (miR-29a) levels are significantly lower in fibrotic livers, as shown with human liver cirrhosis. Such downregulation influences the activation of hepatic stellate cells (HSC). Phosphoinositide 3-kinase p85 alpha (PI3KP85α) is implicated in the regulation of proteostasis mitochondrial integrity and unfolded protein response (UPR) and apoptosis in hepatocytes. This study aimed to investigate the potential therapeutic role of miR-29a in a murine bile duct ligation (BDL)-cholestatic injury and liver fibrosis model. Mice were assigned to four groups: sham, BDL, BDL + scramble miRs, and BDL + miR-29a-mimic. Liver fibrosis and inflammation were assessed by histological staining and mRNA/protein expression of representative markers. Exogenous therapeutics of miR-29a in BDL-stressed mice significantly attenuated glutamic oxaloacetic transaminase (GOT)/glutamic-pyruvic transaminase (GPT) and liver fibrosis, and caused a significant downregulation in markers related to inflammation (IL-1β), fibrogenesis (TGF-β1, α-SMA, and COL1α1), autophagy (p62 and LC3B II), mitochondrial unfolded protein response (UPR^mt^; C/EBP homologous protein (CHOP), heat shock protein 60 (HSP60), and Lon protease-1 (LONP1, a mitochondrial protease), and PI3KP85α within the liver tissue. An in vitro luciferase reporter assay further confirmed that miR-29a mimic directly targets mRNA 3′ untranslated region (UTR) of PI3KP85α to suppress its expression in HepG2 cell line. Our data provide new insights that therapeutic miR-29a improves cholestasis-induced hepatic inflammation and fibrosis and proteotstasis via blocking PI3KP85α, highlighting the potential of miR-29a targeted therapy for liver injury.

## 1. Introduction

Mounting evidence has highlighted persistent liver injury results in liver fibrosis, which involves various cell types. Following a persistent liver injury, hepatic stellate cells (HSCs) are activated and experience morphologic and functional trans-differentiation into contractile myofibroblastic cells [1]. Moreover, activated HSCs are responsible for secretion of profibrogenic mediators, transforming growth factor-β (TGF-β), and generates extracellular matrix (ECM) proteins that can worsen the wound-healing process, including collagen types I/III, fibronectin, and laminin [2]. MicroRNAs (miRNAs) are a class of highly conserved short noncoding RNAs that regulate gene expression at a post-transcriptional level. A considerable decrease of miR-29 family (a to c) in humans with liver cirrhosis and a hepatic fibrosis animal model induced by the administration of hepatotoxin has been reported. Furthermore, their role in affecting HSC activation has been demonstrated. TGF-β can be secreted by hepatocytes, Kupffer cells, and sinusoidal endothelial cells, functioning as an activator for HSC activation [3]. Mechanistically, TGF-β1 causes HSC to activate, transdifferentiate, and secrete ECM through the downregulation of microRNA-29a (miR-29a) [4,5]. Of note, the overexpression of miR-29a in murine HSC has been shown to suppress fibrogenic genes such as collagen-1α1 (COL1α1) and collagen-4α1 [4,5,6], by directly targeting these extracellular matrix genes’ mRNA. However, evidence of whether miR-29a level is implicated in the pathogenesis of acute cholestasis is still scarce and whether exogenous miR-29a exerts therapeutic potential is yet to be investigated.

Recent lines of study suggest that phosphoinositide 3-kinase p85 alpha (PI3KP85α) is involved in the mechanisms in response to aberrant proteotoxic stress in the liver or hepatocytes. Proteotoxic stress occurring in liver diseases such as non-alcoholic fatty liver disease (NAFLD), nonalcoholic steatohepatitis (NASH), and fibrosis can induce ER stress, autophagy, as well as mitochondrial unfolded protein response (UPR^mt^), which is a transcriptional response induced by mitochondrial dysfunction and relayed by a retrograde mitochondria-to-nucleus crosstalk [7,8]. Activation of UPR^mt^ requires phosphorylated eukaryotic translation initiation factor 2 subunit 1 (eIF2α) to reduce global protein synthesis while boosting the expression of transcription factors, including C/EBP homologous protein (CHOP), ATF4, and ATF5. These transcription factors are involved in activating genes functioning to repair the proteotoxic stress, including chaperones and proteases [7]. Furthermore, activation of CHOP plays a central role in regulating inflammation, fibrogenesis, and autophagic flux in the pathogenesis of hepatitis and liver fibrosis [9,10]. In this regard, PI3KP85α is required to relay experimental ER stress and CHOP activation in tunicamycin-or alcohol-stressed liver [11,12]. However, whether miR-29a modifies PI3KP85α expression to exert a therapeutic effect in liver fibrosis is yet to be clarified.

In recent years, our research team has been dedicated to exploring the molecular mechanism of miR-29a in the pathogenesis of liver fibrosis [1,13,14,15,16,17,18,19,20,21]. We have already demonstrated that genetic overexpression of miR-29a exerts a protective effect against hepatic damage and fibrosis via a number of pathways in the cholestatic mouse model [1,15,16,17,18,19,20]. However, evidence of whether miR-29a can exert a therapeutic effect on cholestatic liver damage is still scarce. As for our previous studies and other literature, we hypothesize that the therapeutic exogenous administration of miR-29a via tall-vein injection may mitigate murine bile duct ligation (BDL)-induced cholestatic liver injury and fibrosis through direct targeting PI3KP85α.

## 2. Results

### 2.1. Exogenous miR-29a Injection Significantly Reduces Liver Injury and Fibrosis in the Context of BDL

**Figure 1:**
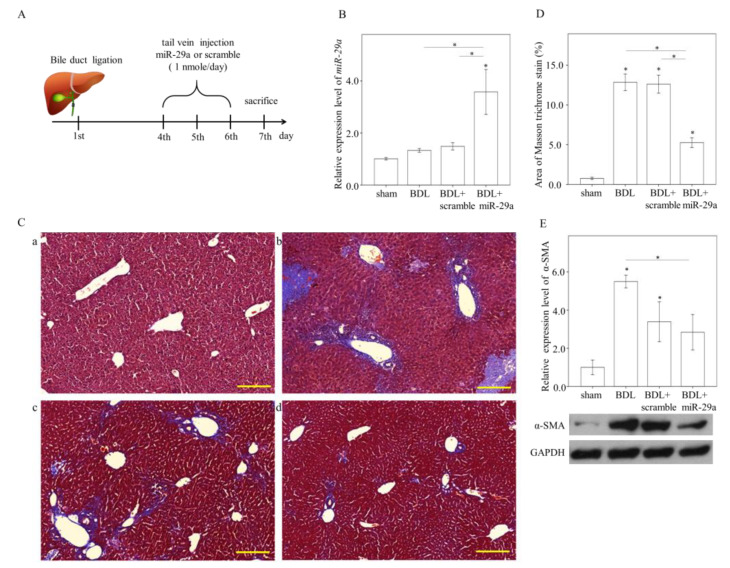
Exogenous miR-29a injection significantly reduces liver fibrosis in the context of BDL. (**A**) Experimental procedure. (**B**) quantitative real-time PCR (qRT-PCR) results of miR-29a levels in liver specimens. N = 6–13. (**C**) Representative image of Masson trichrome staining. a: sham, b: BDL, c: BDL + scramble, d: BDL+miR-29a. Blue stain indicates collagen matrix accumulation. Scale bar, 200 μm(D) quantification results of Masson trichrome staining. Positive staining area (%) was quantified using ImageJ. N = 6–7. (**E**) Representative blotting image and densitometric results of α-SMA protein expression. N = 6 for each group. Histogram data are expressed as mean ± SE. * *p* < 0.05 between the groups. Sham, sham surgery only. BDL, bile duct ligation operation only. BDL + scramble, mice received exogenous scramble injection after BDL. BDL + miR-29a, mice received exogenous miR-29a injection after BDL. α-SMA, alpha-smooth muscle actin.

To develop in vivo delivery via intravenous administration, a polymer-based material in vivo-jetPEI^®^ to condense miR-29a in nanoscale particles was utilized. We firstly performed a pilot experiment that included no-treated (NT) and miR-29a mimic administration at 0,1 or 5 nmole/30 g, showing that 1 nomle caused relatively effective increase in hepatic miR-29a level (*p* = 0.06, Figure S1) and was chosen as administration dose thereafter. Mice were allocated to four groups: sham-operated control, BDL, BDL + scramble, and BDL + miR-29a-mimic. A seven-day experimental flow chart is shown as Figure 1A. BDL per se had no effect on miR-29a expression in the liver, compared with that in sham, while exogenous miR-29a administration increased two to three times compared with other experimental groups (*p* < 0.05, Figure 1B). BDL, BDL + scramble, and BDL + miR-29a presented a decrease in the body weight and liver-to-body percentage, compared with sham group at day 7 (Table 1). BDL + miR-29a showed an increase in body weight gain compared to BDL, but not to BDL + scramble. Both BDL + scramble and BDL + miR-29a showed an increase in liver-to-body ratio (Table 1). Masson trichrome staining used to determine hepatic fibrosis showed that BDL group exhibited more collagen-matrix-accumulated blue staining around the portal area in liver specimens than that of BDL surgery mice, but not in the sham group (*p* < 0.05, Figure 1C–D). This histopathology of fibrosis has been significantly reduced in BDL + miR-29a (*p* < 0.05, compared with BDL and BDL + scramble; Figure 1C–D). Furthermore, alpha-smooth muscle actin (α-SMA) protein expression, which denotes a marker for HSC activation and hepatic fibrosis, was decreased in BDL-miR29a, compared with that in BDL (*p* < 0.05, Figure 1E). These results indicate that exogenous miR-29a injection via tail veil exerts therapeutic effect in ameliorating hepatic inflammation and fibrosis in cholestatic liver.

### 2.2. Exogenous Administration of miR-29a via Tail Vin Injection Significantly Restores the Markers Assessing Hepatic Inflammation and Fibrosis

**Figure 2:**
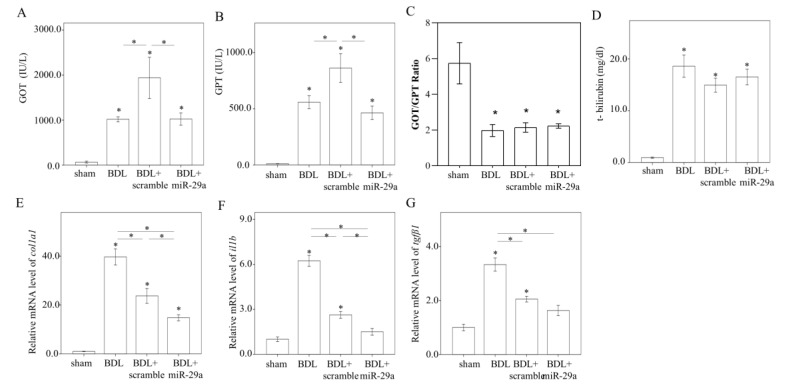
Exogenous miR-29a injection significantly reverses the markers assessing hepatic inflammation and fibrosis. (**A**–**D**) Serum GOT, GPT, GOT/GPT ratio, and total bilirubin. mRNA expression level of (**E**) *colla1,* (**F**) *Il1b,* and (**G**) *tgfb1*. *β-actin* level is used as the normalization control. N = 5–9 was used for each group. Data are expressed as mean ± SE. * *p* < 0.05 between the groups. Sham, a sham surgery only. BDL, a bile duct ligation operation only. BDL + scramble, mice received exogenous scramble injection after BDL. BDL + miR-29a, mice received exogenous miR-29a injection after BDL. GOT: glutamic oxaloacetic transaminase. GPT: glutamic-pyruvic transaminase.

BDL induced hepatic inflammation, as evidenced by an increase in serum GOT, GPT, and total bilirubin level, (*p* < 0.05, Figure 2A–D). BDL + miR-29a presented a lower GOT/GPT level than BDL + scramble (*p* < 0.05, Figure 2A–B), indicating hepatoprotective effect of miR-29a. However, as BDL + scramble showed a higher GOT/GPT value than BDL group (*p* < 0.05, Figure 2A,B), we deduced that an off-target effect derived from exogenous small RNA, which can perturb innate immune response [22], might be involved. On the other hand, BDL, the BDL + scramble, and BDL + miR-29 group showed a lower GOT/GTP ratio than sham group (*p* < 0.05, Figure 2C). Then, we confirmed the expression level of genes corresponding to histological and biochemical manifestations by using qRT-PCR. The mRNA level of inflammatory marker *Il1b,* and fibrogenic markers *colla1* and *tgfb1,* was increased in BDL group, compared with other groups (all *p* < 0.05, Figure 2D–F), and significantly decreased in BDL + miR-29a group (all *p* < 0.05, Figure 2E–G).

### 2.3. Exogenous miR-29a Injection Significantly Reduced PI3KP85α, as well as Molecules Associated with UPR^mt^ and Autophagy in Colestatic Livers

**Figure 3:**
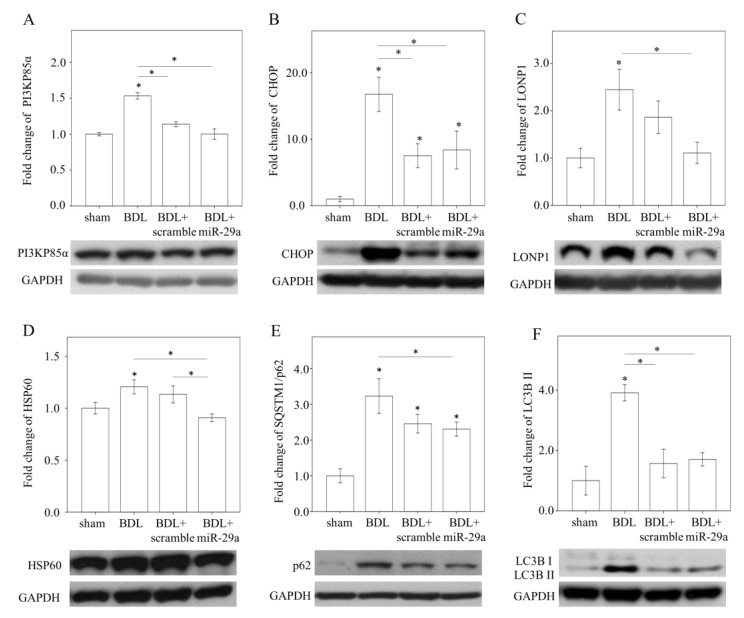
Effect of miR-29a treatment on protein expression of PI3Kp85α and proteostatic molecules in the liver. Representative image of western blot and densitometric results of PI3Kp85α (**A**), CHOP (**B**), LONP1 (**C**) HSP60 (**D**), SQSTM1/p62 (**E**), and LC3B II (**F**), in mice liver. Data collected from N = 5–6 per group are expressed as mean ± SE. * *p* < 0.05 between the groups. Sham, a sham surgery only. BDL, a bile duct ligation operation only. BDL + scramble, mice received exogenous scramble injection after BDL. BDL + miR-29a, mice received exogenous injection of miR-29a mimic after BDL.

Given that PI3KP85α was shown to be central to relay proteostatic signaling in stressed liver [11,12], and is predicted as a target of miR-29a from bioinformatic database (www.mirbase.org), we hypothesize that PI3KP85α may be involved in the mechanism mediating the effect of miR-29a. As shown in Figure 3A, western blot analysis showed that PI3KP85α was induced in BDL, compared with sham group, and was reduced in BDL + miR-29a group (*p* < 0.05). Likewise, CHOP, the putative downstream of PI3KP85α, was increased in BDL, compared with sham group, while reduced in BDL + miR-29a group (*p* < 0.05, Figure 3B). UPR^mt^ effector HSP60 and LONP1, presented corresponding expression manner in line with CHOP (*p* < 0.05, Figure 3C–D). Other proteostatic stress makers p62 and LC3B II were also increased in BDL, compared with sham, while decreased in BDL + miR-29a (*p* < 0.05, Figure 3E,F). These results suggest that miR-29a administration mediates inhibition of PI3KP85α to alleviate proteotoxic stress in cholestatic liver.

### 2.4. miR-29a Acts to Suppress PI3Kp85α Expression via Directly Targeting its 3′UTR

**Figure 4:**
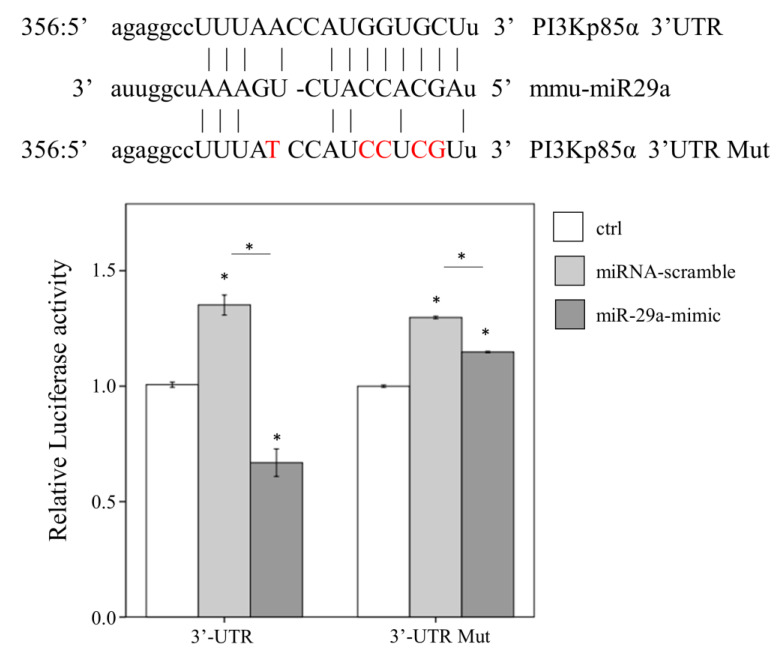
Interaction of miR-29a with the 3′ UTR of PI3Kp85α by luciferase assay. Shown above is the sequence information and mutual binding status of PI3Kp85-3′UTR, mmu-miR29a, and PI3Kp85-3′UTR Mut. The nucleotides in red indicate mismatching sites. HepG2 cells were firstly transfected with PI3Kp85α-3′UTR or PI3Kp85α-3′UTR mutant luciferase reporter plasmid, then treated with control medium (ctrl), miRNA-scramble, or miR-29a mimic, and finally lysed to detect the luciferase signal. Three independent experiments (N = 3) with at least triplicate for each experiment were conducted. Data expressed as mean ± SE. * indicates a *p* < 0.05 between the groups. mmu-miR29a, mouse-origin miR-29a. ctrl, control. Mut, mutant. UTR, untranslated region.

To verify the suppressive activity of miR-29a on mRNA 3′ untranslated region (UTR) of PI3KP85α, we conducted in vitro luciferase reporter assay in human liver hepatocellular carcinoma HepG2 cells harboring PI3Kp85α-3′UTR or PI3Kp85α-3′UTR mutant (Mut) luciferase reporter construct. MiR-29a mimic or control scramble sequence was transfected into HepG2 cells. As shown in Figure 4, miR-29a mimic significantly reduced the luciferase activity in cells harboring wildtype PI3Kp85α-3′UTR, compared with miR-scramble, while the miR-29a mimic-derived suppressive effect diminished in cells harboring the PI3Kp85α-3′UTR Mut (all *p* < 0.05, Figure 4). This result confirms that miR-29a inhibited the expression of PI3Kp85α by targeting its 3′UTR.

## 3. Discussion

**Figure 5:**
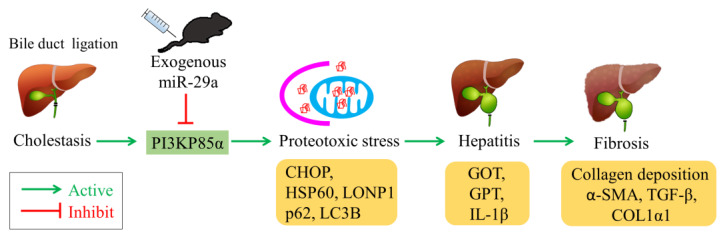
Proposed model of miR-29a-relayed pathway in the treatment of cholestasis. Exogenous miR-29a ameliorates cholestasis-induced proteotoxic stress, hepatitis, and liver fibrosis by suppressing PI3K85α.

In this study, we demonstrated that intravenous miR-29a administration exerts protective effect on hepatic inflammation and fibrosis in cholestatic mouse liver. In addition, we revealed that miR-29a targets and suppresses PI3KP85α expression along with downregulation of proteostatic molecules, including CHOP, HSP60, LONP1, p62, and LC3B II. A proposed model of miR-29a-relayed pathway is depicted in Figure 5.

A decrease of miR-29a abundance has been reported in the liver of patients with cirrhosis and that of mouse treated with chemical hepatotoxin [4,23], which take years and weeks to form pathological manifestation, respectively. In contrast, the present study demonstrates that BDL insult, which sustains over a span of three days, has no effect on hepatic miR-29a level, indicating miR-29a expression manner in the context of acute damage differs from that in relatively chronic situation. Nevertheless, our previous study demonstrates that mice harboring overexpressed miR-29a present ameliorated BDL-induced liver fibrosis [1,19]. Of note, this study further exhibits that exogenous administration of miR-29a exerts hepatic protection, highlighting the potential of miR-29a in new drug development.

In our previous studies, mice with overexpression of miR-29a present alleviated liver damage in the context of cholestasis induced by BDL [1,15,16,18,19]. Nevertheless, these results are not sufficient to extrapolate that miR-29a possesses therapeutic effect, because a C57BL/6 mouse harbors overexpressed miR-29a from birth and is distinct from scenario using treatment approach. In the present study, we sought to condense miR-29a by a polymer-based material and conducted in vivo delivery via intravenous administration and demonstrated its therapeutic effect in counteracting hepatic inflammation and fibrosis (Figure 1 and Figure 2). Although information regarding exogenous miR-29a administration in cholestatic liver is limited, Matsumoto et al. have shown hepatoprotective effect of intravenous delivery of miR-29a in a chronic liver fibrosis model induced by CCl_4_ [23], in line with our observation.

Off-target activity of noncoding RNA such as miR, siRNA and shRNA, through activation of some types of Toll-like receptors, can complicate the interpretation of phenotypic results and may lead to unexpected toxicity to the cells [22]. In our study, scramble miR administration causes an increase in serum GOT and GPT level (BDL vs. BDL + scramble, Figure 1B), raising concern about off-target effect. However, scramble miR is nondetrimental in some parameters, including having no influence on liver fibrosis (BDL vs. BDL + scramble, Figure 1C), a decrease in liver-to-body weight (BDL vs. BDL + scramble, Table 1), and reduced level in markers related to inflammation and fibrogenesis (BDL vs. BDL + scramble, Figure 2B–D). Importantly, miR-29a mimic per se has no detrimental effect on all parameters. Nevertheless, further study is warranted and should take safety, and toxicity issue of miR-based approach, into consideration.

Our previous study has demonstrated that the ER stress is involved in the pathogenesis of BDL-induced liver fibrosis, and its provocation can be alleviated in mice harboring miR-29a transgene [18]. Of note, the mutual interaction between mitochondria and ER regulates proteostasis and cellular stress by the activity of mitochondrial-localizing proteins and interorganellar communication [24,25]. In the present study, induced CHOP, which functions as a key mediator of both ER stress and UPR^mt^, is suppressed by miR-29a mimic, implying that not only mitochondria but also ER stress may be affected in this scenario. Further study is warranted to elaborate on the role of miR-29a on ER stress and ER-mitochondria crosstalk in the context of cholestasis.

Several lines of study have suggested a deteriorative role of PI3KP85α in the development of liver fibrosis. In vitro studies demonstrated that pharmacological inhibition of PI3KP85α exerts an antifibrogenic effect in human HSC and liver tissue [26,27]. Son et al. presented that PI3K inhibition by adenovirus-based delivery attenuates in vitro fibrogenic activity in HSC and in vivo hepatic fibrosis, while fails to ameliorate hepatic inflammation [28], indicating specific inhibition to PI3K likely not effective in liver injury. In fact, potential hepatoxicity of PI3K inhibitor has been noted [29]. In this regard, our study demonstrates that intravenous administration of miR-29a exerts inhibitory effect of PI3KP85α, and significant improvement in both hepatic inflammation and fibrosis. As such, miR-based exogenous approach appears to be superior. Nevertheless, the exact mechanism needs to be clarified in future study.

The consequence of PI3K inhibition in the hepatocytes results in suppression of CHOP activity, leading to impaired mitochondrial homeostasis [12]. Furthermore, CHOP along with HSP60 and LONP1 constituting UPR^mt^ are central to modulating mitochondrial proteostasis [7]. Abrogation of CHOP was shown to mitigate hepatic inflammation and fibrosis as well as proteotoxic response [9,30]. In the context of NASH/NAFLD, provoked autophagic activity is suppressed by CHOP silencing or deletion [10]. Herein, our study demonstrates that miR-29a-supresses PI3KP85a to exert inhibitory effect on CHOP, which may contribute to a lessened mitochondrial proteotoxic status, as evidenced by reduced HSP60 and LONP1 as well as p62 and LC3B II (Figure 3). Although the molecular mechanism that controls miR-29a/PI3KP85a/CHOP involvement in the stressed liver is still not clear, a recent study suggests an emerging role of miRs in preserving the hepatic mitochondrial proteostasis [31].

## 4. Materials and Methods

### 4.1. Ethics Statement

Our animal protocol was reviewed and approved by the Institutional Animal Care and Use Committee (IACUC) of Chang Gung Memorial Hospital (19 December 2017; Approval number: 2017091801). We purchased C57BL/6, 7-week-old mice from BioLASCO Taiwan Co., Ltd. (Taipei, Taiwan) All animals were housed in an animal facility at 22 °C, with a relative humidity of 55%, in a 12 h light/12 h dark cycle, with food and sterile tap water available ad libitum.

### 4.2. Animal Model and Experimental Protocol

Six to eight mice were used for all our experiments. The operation procedure of common bile duct ligation or sham control was described in a previous study [1]. MiR-29a mimic (UAGCACCAUCUGAAAUCGGUUA; #C-300504-07-0050, Horizon Discovery) and scramble control sequence (UCACAACCUCCUAGAAAGAGUAGA; #CN-001000-01-50, respectively, purchased from Horizon Discovery) used for the purpose of in vivo delivery were dissolved in 200 μL of in vivo-jetPEI^®^ reagent (201-50G, Polyplus-transfection) in accordance with the manufacturer’s standard protocol. Surgical operation was conducted at the 1st day. From the 4th to 6th day, the BDL group was allocated to two groups of tail vein injection with scrambled (BDL + scramble, 1 nmole/30 g) or miR-29a-mimic (BDL + miR-29a, 1 nmole/30 g), one shot per day, three shots in total. Anthropometric measurements were conducted at the beginning and end of the study. All the mice were euthanized 1 week postoperatively. Liver tissues were dissected, snap-frozen, and processed, to isolate total RNA and proteins. All specimens were stored at −80 °C until the biochemical analysis was carried out.

### 4.3. Histological Analysis

Fresh livers were fixed in in 10% formaldehyde and embedded in paraffin. Five micrometer sections were subjected to Masson trichrome stain (Poly Scientific, Bay Shore, NY, USA) in accordance with the manufacturer’s standard protocol, except for the 90 min incubation of aniline blue-solution I. The prolonged incubation was an optimized condition in our laboratory. The quantification of staining signal was analyzed by independent color channel of ImageJ (version 1.48, Wayne Rasband, National Institutes of Health, Rockville, MD, USA).

### 4.4. Quantitative Real-Time PCR (qRT-PCR)

Total RNA was extracted by using TRIzol^®^ reagent (Invitrogen, Carlsbad, CA, USA) from liver tissue and used to generate cDNA with an oligodeoxynucleotide primer (oligo dT15) according to the manufacturer’s protocol (Promega, Madison, WI). MicroRNA Isolation Kits (BioChain Institute, Inc, Hayward, CA, USA) was used for to isolate total microRNA. The qPCR reaction of COL1α1, IL-1β, and tgf-β as well as normalization control glyceraldehyde 3-phosphate dehydrogenase (GAPDF)was conducted with 2X SYBR Green PCR Master Mix (Roche Molecular Systems, Inc., Pleasanton, CA, USA) on LightCycler480^®^ (Roche). Each PCR reaction included 5 μM forward and reverse primers and the cDNA product diluted at 8× in a total reaction volume of 10 μL. For qPCR reaction of COL1α1, IL-1β and *tgf-β*, an initial amplification was done with a denaturation step at 95 °C for 10 min, followed by 45 cycles of denaturation at 95 °C for 30 s, primer annealing at 62 °C for 15 s, and primer extension at 72 °C for 25 s followed by melting curve analysis. The primer sequences were as follows. COL1α1: Forward sequence 5′-CTGGCAAGAATGGCGAC-3, reverse sequence 5′-CCCTGGAGACCAGAGAAG-3′; IL-1β: forward sequence 5′-GAGGACATGAGCACCTTCTTT-3′, reverse sequence 5′-GCCTGTAGTGCAGTTGTCTAA-3′; TGF-β1: forward sequence 5′-GTGGACCGCAACAACGCCATCT-3′, reverse sequence 5′-GCAATGGGGGTT CGGGCACT-3′; GAPDF: forward sequence 5′-GCACAGTCAAGGCCGAGAAT-3′, reverse sequence 5′-GCCTTCTCCATGGTGGTG-3′. The PCR efficiency of COL1α1, IL-1β, TGF-β1, and GAPDF is 1.913, 1.978, 1.981, and 1.964, respectively (Figure S2). For detection of miR-29a expression, predesigned primer/probes for miR-29a (#002112, ThermoFisher) and normalization control sno202 (#001232, ThermoFisher) were used.

### 4.5. Western Blotting

Forty microgram proteins extracted from the liver was separated in 8–15%SDS-PAGE, then transferred onto PVDF membrane and incubated with primary antibodies at 4 °C overnight. The primary antibodies included PI3Kp85α (1:5000; 60225-1 lg, PROTEINTECH, IL, USA), CHOP (1:1000; #2895, cell signaling, MA, USA), SQSTM1/p62 (1:5000; GTX111393, GeneTex, CA, USA), HSP60 (1:5000; sc-1052, Santa Cruz, CA, USA), LONP1 (1:2000; 15440-1-AP, Proteintech, IL, USA), LC3B II (1:5000; #2775, cell signaling, MA, USA), and α-SMA (1:1000; ab5694, abcam, Cambridge, UK). GAPDH (1:100,000; 60004-1 lg, PROTEINTECH, IL, USA) was used for probing protein loading control. After washing twice with TBST solution, PVDF membrane was incubated with secondary antibodies such as horseradish peroxidase-coupled antirabbit immunoglobulin-G antibodies (1:5000; NEF812001EA, PerkinElmer, MA, USA) or HRP antimouse immunoglobulin-G antibodies (1:10,000; NEF822001, PerkinElmer) at room temperature for 1 h. The blots were developed with an ECL Western blotting detection and analysis system (Amersham Pharmacia Biotech, Uppsala, Sweden) and exposed them to film. The signals were quantified by using Quantity One^®^ 1-D analysis software (Bio-Rad Laboratories).

### 4.6. Luciferase Reporter Assay

The wild type pMIR-PI3Kp85α luciferase plasmid was constructed by cloning mouse PI3Kp85α-3′UTR sequence into the pMIR-REPORT™ miRNA Expression Reporter Vector (Applied Biosystems, Foster City, CA, USA), while the pMIR-PI3Kp85α-Mut luciferase plasmid was substituted with five mismatched sites (Figure 4). The plasmids were purified using EasyPrep EndoFree Maxi Plasmid Extraction Kit (BIOTOOLS, Ltd., New Taipei, Taiwan). 9 × 10^5^ HepG2 cells were seeded at a 6 cm culture dish. After 24 h, 3 µg of pMIR-PI3Kp85α luciferase plasmid or pMIR-PI3Kp85α-Mut plasmid was introduced by TurboFect reagent (Thermo Fisher Scientific, Rockford, IL, USA). After another 24 h, 25 nM of miR-29a precursor (mimic-miR-29a, GE Healthcare Dharmacon, IN, USA) or miR control sequence (GE Healthcare Dharmacon) were introduced by using Lipofectamine™ RNAiMAX Transfection Reagent (Invitrogen) as per the manufacturer’s standard protocol. After incubated for 24 h at 37 °C, the cells were lysed for the detection of luciferase signal with Neolite Reporter Gene Assay System (PerkinElmer, Waltham, MA, USA).

### 4.7. Statistical Analysis

Data collected from at least three independent experiments are expressed as mean ± standard error. Statistical significance between groups was analyzed by one-way analysis of variance (ANOVA), followed by the least significant difference (LSD) test for post-hoc testing. Significant level was set at *p* < 0.05. Graph drawing and statistical analysis were conducted with IBM SPSS Statistics V22.0.

## 5. Conclusions

Our findings demonstrate that intravenous administration of miR-29a renders hepatoprotection to cholestatic liver and alleviation of proteotoxic loading by targeting PI3KP85α. Therefore, our results suggest that miR-29a mimic could serve as a possible therapeutic tool to improve the treatment of liver inflammation and fibrosis.

## Figures and Tables

**Table 1:** Anthropometric measurements of the animals.

Parameter	Time Point	Sham	BDL	BDL + Scramble	BDL+miR-29a
Body Weight (g)	Day 0	23.25 ± 0.51	23.26 ± 0.29	24.23 ± 0.32	24.46 ± 0.35
	Day 7	23.93 ± 0.53	18.72 ± 0.35 *^a^*	18.77 ± 0.23 *^a^*	18.82 ± 0.27 *^a^*
Body Weight Gain (%)	Day 7	2.9 ± 0.52	−19.48 ± 1.17 *^a^*	−22.52 ± 0.96 *^a^*	−22.97 ± 1.39 *^a,b^*
Liver Weight (g)	Day 7	1.19 ± 0.07	1.36 ± 0.04 *^a^*	1.21 ± 0.06	1.22 ± 0.05
Liver/Body Weight (%)	Day 7	4.94 ± 0.21	7.25 ± 0.13 *^a^*	6.44 ± 0.26 *^a,b^*	6.49 ± 0.18 *^a,b^*

Data collected from seven to ten mice each group expressed as mean ± SE; *^a^*, *p* < 0.05 versus sham; *^b^*, *p* < 0.05 versus BDL. BDL: bile duct ligation.

## Supplementary Materials

The following are available online at https://www.mdpi.com/1422-0067/21/10/3636/s1, **Figure S1.** Dose optimization of miR-29a mimic, **Figure S2.** PCR efficiency of *col1α1*, *il-1β*, *tgf-β1*, and *gapdh*.
