# Supplementary material for: Exogenous Therapeutics of Microrna-29a Attenuates Development of Hepatic Fibrosis in Cholestatic Animal Model through Regulation of Phosphoinositide 3-Kinase p85 Alpha
Source: Int J Mol Sci. 2020 May 21;21(10):3636. doi: 10.3390/ijms21103636 (PMC7279217; doi:10.3390/ijms21103636)
Supplement: Supplementary file 1 [file ijms-21-03636-s001.pdf]

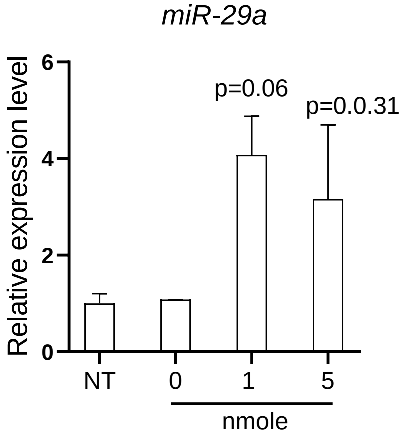

Figure S1. Dose optimization of miR-29a mimic. Mice were categorized into four groups: no-treated (NT), mock, 1 and 5 nomle/30g. Each group includes two animals. Treated groups were individually subjected to administration of 0, 1, and 5 nmole miR-29a mimic dissolved in 200 μl of *in vivo*-jetPEI® through tail vein one shot per day, three days in total. Hepatic *miR-29a* level was determined using qRT-PCR. *Sno202* level was used as normalization control.

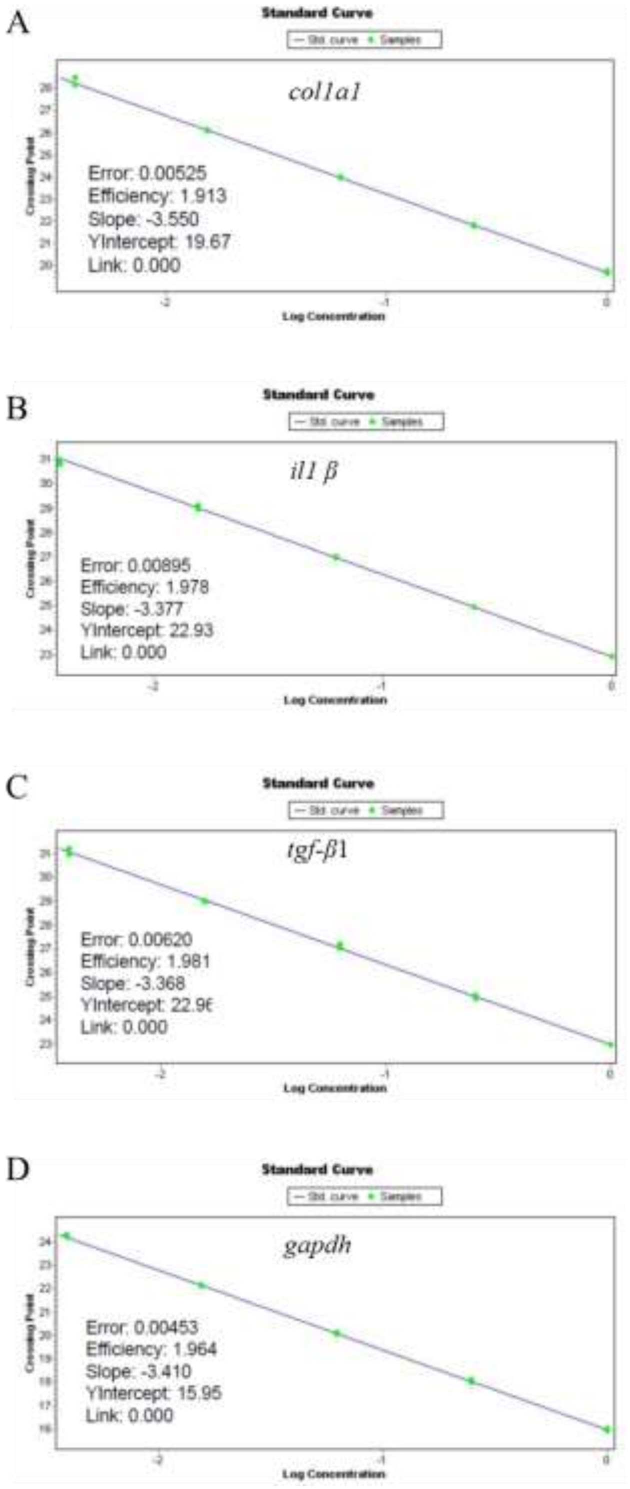

Figure S2. PCR efficiency of *col1α1*, *il-1β*, *tgf-β1*, and *gapdh*. The qRT-PCR reaction was performed with serially diluted cDNA of liver tissue in the presence of designed primers and SYBR green master mix. The Ct value was used for calculation of PCR efficiency of *col1α1*(A), *il-1β*(B), *tgf-β1*(C), and *gapdh* (D).
